# Etiology of bronchiectasis in Qatar: A retrospective study

**DOI:** 10.5339/qmj.2024.qitc.23

**Published:** 2024-03-25

**Authors:** Tayseer Ibrahim, Merlin Thomas, Ibrahim Rasheed, Hassan Mobayed, Hisham Abdul Sattar, Abdullatif Al-Khal, Maryam Al-Nesf

**Affiliations:** Allergy and Immunology Division, Department of Medicine, Hamad Medical Corporation, Doha, Qatar Email: tibrahim2@hamad.qa; Pulmonary Division, Department of Medicine, Hamad Medical Corporation, Doha, Qatar; Communicable Disease Center, Hamad Medical Corporation, Doha, Qatar

**Keywords:** Bronchiectasis, Etiology, TB, Qatar

## Introduction

Bronchiectasis is a heterogeneous disease with an unpleasant socioeconomic burden with different prevalence and underlying etiology in different geographical regions and targeted populations. Tuberculosis was and is still considered one of the major causes of bronchiectasis. However, data from different regions should provide a better understanding of how bronchiectasis develops, especially in Middle Eastern countries with low to moderate tuberculosis prevalence. Many Middle East countries, including Qatar, lack local data on bronchiectasis.

This study aimed to investigate the causes of bronchiectasis in Qatar.

## Methods

A retrospective chart review was conducted in patients diagnosed with bronchiectasis and admitted to Hamad General Hospital (November 2012–September 2018). Inclusion criteria were patients aged 12 years and above with physician-diagnosed bronchiectasis. Data on demographics, underlying causes, and related laboratory/radiology findings were collected.

## Results

A total of 498 patient records were identified, of which only 284 met the inclusion criteria. Most were males (53.8%, 153/284), and the mean age was 48.6 ± 20.8 years. The majority were Arabs (60.2%, 171/284), and 56.7% (97/171) were Qatari. The etiological landscape of bronchiectasis revealed a significant proportion of idiopathic bronchiectasis (44.7%). Notably, previous pulmonary tuberculosis emerged as a cause in 17.3% of bronchiectasis, which highlights the impact of tuberculosis on respiratory health in this population. Cystic fibrosis bronchiectasis, interstitial lung disease, and chronic obstructive pulmonary disease (COPD) were among the identified causes, reflecting the diverse etiological spectrum. The causes of bronchiectasis are summarized in Table 1.

## Conclusion

The current study is the first to report the etiology of bronchiectasis in Qatar. The high prevalence of idiopathic cases indicates the need for further investigation to examine the factors contributing to bronchiectasis in general.

## Conflict of Interest

The authors have no conflict of interest to declare.

## Figures and Tables

**Table 1.** Documented possible causes of bronchiectasis (total no. 284).

**Etiology of bronchiectasis**	**Frequency (%)**
Idiopathic bronchiectasis	127 (44.7)
Pulmonary tuberculosis	49 (17.3)
Cystic fibrosis	27 (9.5)
Interstitial lung disease	19 (6.7)
Chronic obstructive airway disease	15 (5.3)
Primary immune deficiency	10 (3.5)
Connective tissue disease/eosinophilic granulomatosis with polyangiitis	7 (2.5)
*Allergic bronchopulmonary aspergillosis*	7 (2.5)
*Ciliary dyskinesia/Kartagener syndrome*	7 (2.5)
Foreign body/obstructive lesion	6 (2.1)
*Aspiration*	4 (1.4)
*Mounier–Kuhn syndrome*	1 (0.4)
*Cystic lung disease*	1 (0.4)
*Homocystinaemia*	1 (0.4)
*Ehlers–Danlos syndrome*	1 (0.4)
*Congenital bronchial stenosis*	1 (0.4)
*Spinal muscular atrophy*	1 (0.4)

